# Ectopic expression of the WWOX gene suppresses stemness of human ovarian cancer stem cells

**DOI:** 10.3892/ol.2015.2971

**Published:** 2015-02-17

**Authors:** HONG CHAO YAN, JUN XU, LI SHA FANG, YING YING QIU, XIAO MAN LIN, HONG XIANG HUANG, QIU YU HAN

**Affiliations:** Department of Obstetrics and Gynecology, The Affiliated Hospital of Xuzhou Medical College, Xuzhou, Jiangsu 221002, P.R. China

**Keywords:** WW domain-containing oxidoreductase gene, culture, ovarian cancer, cancer stem cell

## Abstract

The present study aimed to investigate the effects of the WW domain-containing oxidoreductase (*WWOX)* gene on the stem cell properties of human ovarian cancer stem cells. A eukaryotic expression vector containing the *WWOX* gene was transfected into human ovarian cancer stem cells and Western blotting was used to assess the expression of WWOX protein in the transfected cells compared with the control cells (untransfected cells and cells transfected with the empty vector). The self-renewal abilities of these three types of stem cells was also assessed *in vitro*. To monitor changes in their differentiation potential, cells were cultured in medium supplemented with serum, and the expression of specific stem cell markers was determined. Drug-sensitivity tests were used to measure the sensitivity of the stem cells to cisplatin, doxorubicin, and mitoxantrone. The cells were also transplanted into non-obese diabetic (NOD)/severe combined immunodeficiency (SCID) mice to determine the changes in their tumorigenicity *in vivo*. Cells transfected with the WWOX-expressing plasmid stably expressed WWOX protein, while no WWOX protein was detected in control cells. Compared with the two types of control cells, WWOX-expressing stem cells manifested significantly reduced self-renewal ability. Compared with control cells, the expression levels of stem cell markers, including CD133, CD117, ATP-binding cassette sub-family G member 2, Nanog, octamer-binding transcription factor 4 and breast cancer resistance protein, were significantly lower in WWOX-expressing cells, while the level of the differentiation marker E-cadherin was significantly higher in WWOX-expressing cells. Furthermore, WWOX-expressing cells were more sensitive to treatment with cisplatin, doxorubicin and mitoxantrone. In NOD/SCID mice, the tumorigenicity of WWOX-expressing cells was significantly lower compared with that of control cells. The results indicate that the tumor suppressor WWOX suppresses stem cell properties in cancer stem cells, including self-renewal ability, differentiation potential, *in vivo* tumorigenic capability, high-level expression of stem cell genes and multidrug resistance.

## Introduction

The cancer stem cell theory proposes that cancer is a stem cell disease, where tumor recurrence is caused by cancer stem cells. Cancer stem cells are tumorigenic cells that possess self-renewal ability, have unlimited proliferative potential, and can differentiate into multiple cell types. These cells are hypothesized to be the cause of tumor initiation, abnormal proliferation, invasion, metastasis, drug resistance and recurrence (1–3). Previously, our group cultured HO8910 cells (a cell line from a poorly-differentiated human ovarian serous cystadenocarcinoma) in suspension with serum-free medium supplemented with paclitaxel, and isolated CD133+, CD117+ cells, which were determined to be cancer stem cells by a series of *in vitro* and *in vivo* experiments (4). These cells provide a valuable tool with which to study the biological characteristics of ovarian cancer stem cells.

The tumor suppressor gene WW domain-containing oxidoreductase (*WWOX)* was isolated by Bednarek *et al* (5) using shot-gun sequencing. WWOX is a 414-amino acid protein, with two WW structural domains at its amino-terminus. The WW domain mediates protein-protein interactions, which is essential for the signaling pathways used by tumor suppressors to inhibit tumor growth. The WWOX protein localizes to the mitochondria under normal conditions but, in response to stress stimuli, the synthesis of this protein increases, mitochondrial permeability is altered, and WWOX translocates to the nucleus where it regulates gene expression. WWOX may inhibit tumor initiation and progression through multiple signaling pathways, including the following: Tumor necrosis factor receptor type 1-associated DEATH domain protein and tumor necrosis factor receptor-associated factor 2-mediated apoptosis pathways; c-Jun N-terminal kinase 1-mediated stress response pathways; and p53-initiated apoptotic pathways (6,7). In addition, our previous studies have demonstrated that WWOX alters the biological phenotype of ovarian cancer stem cells, and is important in the formation and progression of ovarian cancer (8–10).

In the current study, a *WWOX* gene-containing eukaryotic expression vector was introduced into ovarian cancer stem cells by transfection, in order to evaluate the effects of WWOX on the stem cell properties of these cells.

## Materials and methods

### Materials

Human ovarian cancer stem cells were isolated and stored in the Laboratory of Obstetrics and Gynecology at The Affiliated Hospital of Xuzhou Medical College (Xuzhou, China). These cells have previously been confirmed to possess stem cell properties, including self-renewal ability, differentiation potential, *in vivo* tumorigenic capability, high-level expression of stem cell genes and multidrug resistance (4). The pcDNA3.1-*WWOX* eukaryotic expression vector was constructed by and stored in the same laboratory (11). The pcDNA3.1 empty plasmid was provided by Professor Shuqun Hu at the Molecular Biology Research Center of Xuzhou Medical College. Serum-free medium, supplemented with epidermal growth factor (EGF), basic fibroblast growth factor (bFGF), Noggin and leukemia inhibitory factor (LIF), was purchased from Sigma-Aldrich (St. Louis, MO, USA). Primary antibodies to CD133 (cat. no. MAB4310), CD117 (cat. no. MA1-12192), ATP-binding cassette sub-family G member 2 (ABCG2; cat. no. AM1125a), Nanog (cat. no. AP1486c), octamer-binding transcription factor 4 (OCT4; cat. no. NRG1.1), breast cancer resistance protein (BCRP; cat. no. 254515) and E-cadherin (cat. no. MA5-12547) were purchased from Chemicon (Billerica, MA, USA). Cisplatin, doxorubicin and mitoxantrone were purchased from XinYu Biotechnology (Shanghai, China). Female non-obese diabetic (NOD)/severe combined immunodeficiency (SCID) mice (4–6 weeks of age) were purchased from the Chinese Academy of Sciences Shanghai Laboratory Animal Center (Shanghai, China). All animal studies were approved by the Ethics Committee of The Affiliated Hospital of Xuzhou Medical College. Written informed consent was obtained from the patient.

### Cell culture

Human ovarian cancer stem cells were cultured in serum-free medium supplemented with EGF, bFGF, Noggin, and LIF at 37°C in a humidified incubator with 5% CO_2_ in compressed air.

### Gene transfection

Plasmids were transfected into ovarian cancer stem cells using the Lipofector Liposomal Transfection Kit (Beyotime Institute of Biotechnology, Shanghai, China), following the manufacturer’s instructions (transfection efficiency, 68%), and stably transfected cells were subsequently isolated and expanded in culture. A eukaryotic expression vector containing the *WWOX* gene (pcDNA3.1-*WWOX*) was used to produce WWOX expression. Cells transfected with the empty vector (pcDNA3.1) and untransfected cells were used as controls.

### Western blot assay

The three types of cells (*WWOX*-transfected, empty vector-transfected and untransfected) were harvested during the growth phase and lysed on ice in 200 μl lysis buffer. Protein concentrations were determined using a bicinchoninic acid assay. Proteins were separated by 10% sodium dodecyl sulfate-polyacrylamide gel electrophoresis, and transferred onto nitrocellulose membranes. Membranes were blocked with 5% non-fat dry milk for 60 min, incubated with rabbit-anti-human WWOX antibodies (dilution, 1:1,000; cat. no. 15800667461; Hangzhou Sijiqing Biology Engineering Materials Co., Ltd., Hangzhou, China) at 4°C overnight, and subsequently incubated with horseradish peroxidase-conjugated goat-anti-rabbit antibodies (dilution, 1:10,000; cat. no. 13764022678; Sigma-Aldrich) at room temperature for 2 h. Membranes were developed with enhanced chemiluminescence reagents (Hangzhou Sijiqing Biology Engineering Materials Co., Ltd.), and exposed to high sensitivity X-ray films in the dark.

### Analysis of self-renewal abilities of ovarian cancer stem cells

Cells were dissociated into single cells suspensions using 0.25% trypsin, and the number of viable cells was counted following trypan blue staining. Cells were subsequently diluted to a density of 1×10^3^ cells/ml in serum-free medium, and plated into 96-well plates at 100 μl/well; thus each well contained 100 cells. Each cell type was plated into 20 wells, and 100 μl medium was added to each well, prior to incubation in a humidified incubator at 37°C in an atmosphere of 5% CO_2_ for 48 h. An additional 25 μl of medium was added to each well every subsequent day, and the cells were observed each day for sphere formation. Following an incubation period of eight days, the number of cell spheres in each well was counted under a microscope.

### Analysis of cell differentiation

Stem cells were resuspended in RPMI1640 medium containing 10% fetal bovine serum, and allowed to adhere to the plate and differentiate. Cell differentiation was observed under a microscope. Expression of CD133+ and CD117+ on the cells prior to and following differentiation was analyzed by flow cytometry, while the expression of ABCG2, Nanog, OCT4, BCRP, and E-cadherin was analyzed by Western blot analysis.

### Drug-sensitivity test

Cell suspensions were stained with trypan blue to count the number of viable cells, plated into 96-well plates at 6,000 cells/well, and cultured in serum-free medium in the presence of one of the three drugs to be tested (cisplatin, doxorubicin and mitoxantrone). Each drug was tested at two concentrations near its half maximal inhibitory concentration (IC_50_): Cisplatin, 0.25 and 0.5 μg/ml; doxorubicin, 0.5 and 1.5 μmol/l; and mitoxantrone, 0.05 and 0.25 μg/ml. Five replicate wells were prepared for each condition. Following a 48 h incubation, the medium was removed, and 10% water-soluble tetrazolium salt-8 (Pfizer Pharmaceutical Co., Ltd., Billerica, MA, USA) was added to the wells for measurement. The CCK-8 reagent was also added to wells without cells as blanks. The plates were placed in a humidified incubator, with an atmosphere of 5% CO_2,_ for 2 h at 37°C, and the absorbance (A) at 450 nm was measured with an ultraviolet spectrophotometer. The cellular viability following drug treatment was compared with the same type of cells without drug treatment, and relative viability was calculated as (A_drug treated_ − A_blank_) / (A_untreated_ − A_blank_).

### In vivo tumorigenesis assay

Cell suspensions were stained with trypan blue to count the number of viable cells. Various quantities of cells (2×10^5^, 2×10^4^, 2×10^3^ or 1×10^3^) were subsequently injected subcutaneously into the right axilla of female NOD/SCID mice. The 15 mice at 4–6 weeks of age were randomly divided into three groups, with five mice per group. Mice were housed in a pathogen-free room with controlled temperature (25–27°C), controlled humidity (45–50%) and filtered fresh air, and received sterile food and water *ad libitum*. Tumor growth was recorded twice per week. Tumorigenicity was represented as tumorigenesis rate (number of mice that developed tumors / total number of mice that received cells) and tumor-forming time (the time from cell inoculation until the tumors became palpable). When tumors reached 1 cm^3^ in size, or at three months following cell transplantation if no tumor was formed, mice were sacrificed. All procedures were conducted in accordance with animal research ethical guidelines. This study was approved by the ethics committee of The Affiliated Hospital of Xuzhou Medical College.

### Statistical Analysis

All data are presented as mean ± standard deviation (SD). Statistical analysis was performed using SPSS version 13.0 software (SPSS, Inc., Chicago, IL, USA). Analysis of variance was used for intergroup comparisons, while Tukey’s test was used for pairwise comparisons between multiple groups. P<0.05 was considered to indicate a statistically significant difference.

## Results

### Ectopic expression of WWOX protein in ovarian cancer stem cells by gene transfection

**Figure 1:**
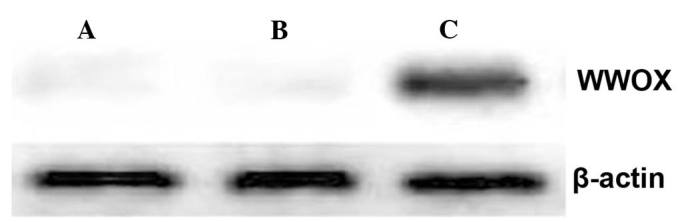
WWOX protein and β-actin (positive control) expression in ovarian cancer stem cells as analyzed by Western blotting. (A) Untransfected cells; (B) empty vector-transfected cells; (C) WWOX-expressing plasmid transfected cells. WWOX, WW domain-containing oxidoreductase.

Western blot analysis demonstrated that cells transfected with the WWOX-expression plasmid produced high levels of WWOX protein, while it was undetectable in untransfected cells, or cells transfected with the empty vector (Fig. 1).

### WWOX inhibits the self-renewal ability of ovarian cancer stem cells

**Figure 2:**
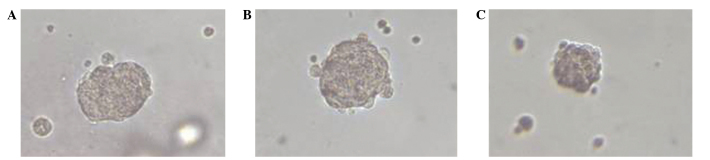
WWOX inhibits the self-renewal ability of ovarian cancer stem cells. (A) Untransfected cells; (B) empty vector-transfected cells; (C) WWOX-expressing cells. WWOX, WW domain-containing oxidoreductase.

A sphere-forming assay was utilized to determine the effect of WWOX expression on the self-renewal ability of ovarian cancer stem cells. Untransfected stem cells and cells transfected with the empty vector began to form spheres following three days in culture, and reached a plateau at seven days. By contrast, WWOX-expressing cells began to form spheres at six days, and reached a plateau at 10 days. Furthermore, WWOX-expressing cells formed significantly fewer cell spheres/well (mean ± SD, 7.61±2.02) (P=0.019) in serum-free medium compared with the cells transfected with the empty vector (mean ± SD, 32.82±3.29) or untransfected cells (mean ± SD, 35.74±2.98). No significant difference was observed between empty vector-transfected cells and untransfected cells. In addition, the largest spheres formed by WWOX-expressing cells were smaller in size compared with those formed by empty vector-transfected cells or untransfected cells (Fig. 2).

### WWOX reduces the differentiation potential of ovarian cancer stem cells

**Figure 3:**
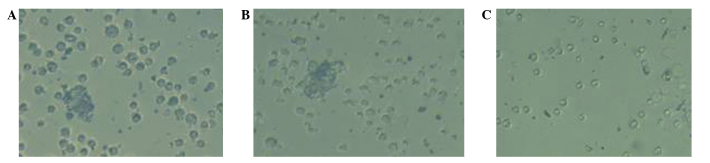
Effects of WWOX on the differentiating capabilities of ovarian cancer stem cells. When plated in a serum-containing medium to allow for cellular differentiation, WWOX-expressing cells began to attach to the culture plate surface after 2 h and almost all cells had adhered to the culture plates. Untransfected cells and empty vector-transfected cells, by contrast, displayed a delay in attachment to the culture plate surface: A number of cells adhered to the culture plates after 6 h, whilst others maintained a spherical morphology and did not become adherent until 24 h. (A) Untransfected cells (6 h); (B) empty vector-transfected cells (6 h); (C) WWOX-expressing cells (2 h). Magnification, ×40. WWOX, WW domain-containing oxidoreductase.

When plated in serum-containing medium to allow for cellular differentiation, WWOX-expressing cells began to attach to the culture plate surface after 2 h and almost all cells had adhered to the culture plates at 12 h. Untransfected cells and empty vector-transfected cells, by contrast, displayed a delay in attachment to the culture plate surface: A number of cells adhered to the culture plates after 6 h, whilst others maintained a spherical morphology and did not become adherent until 24 h (Fig. 3).

The expression of two stem cell markers, CD133 and CD117, was analyzed in these cells using flow cytometry. The percentages of CD133+ and CD117+ populations in the WWOX-expressing cells were 21.3% and 23.4%, respectively, prior to cellular differentiation, and 19.9% and 21.1%, respectively, following differentiation; this difference was not significant. In cells transfected with the empty vector, the percentages of CD133+ and CD117+ cells prior to differentiation were 76.4% and 81.8%, respectively, and decreased significantly following differentiation (25.3% and 28.3%, respectively). Similarly, untransfected cells comprised 79.7% CD133+ cells and 78.5% CD117+ cells prior to differentiation, decreasing significantly to 23.1% and 21.5%, respectively, following differentiation.

**Figure 4:**
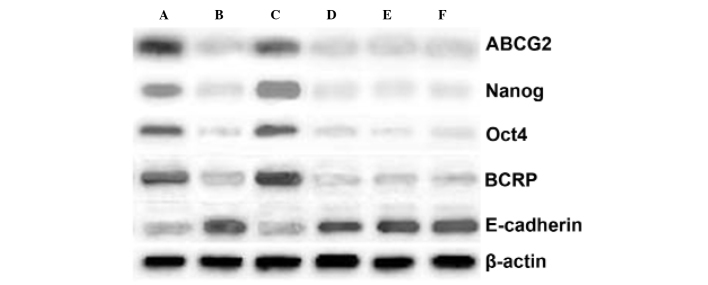
WWOX affects the expression of stem cell markers and E-cadherin in ovarian cancer stem cells. (A) Untransfected cells (before differentiation); (B) untransfected cells (after differentiation); (C) empty vector-transfected cells (before differentiation); (D) empty vector-transfected cells (after differentiation); (E) WWOX-expressing cells (before differentiation), (F) WWOX-expressing cells (after differentiation). WWOX, WW domain-containing oxidoreductase; ABCG2, ATP-binding cassette sub-family G member 2; Oct4, octamer-binding transcription factor 4; BCRP, breast cancer resistance protein.

The protein levels of the stem cell markers ABCG2, Nanog, OCT4, and BCRP in these cells were also analyzed by Western blotting. Protein levels were quantified as follows: The target belt and β-actin protein band gray values were detected and the ratio of target belt/β-actin indicated the relative expression level of the target proteins. In WWOX-expressing stem cells prior to differentiation, the mean ± SD levels of these four proteins were 0.38±0.11, 0.42±0.07, 0.31±0.03, and 0.37±0.13, respectively, and did not differ significantly following differentiation (0.34±0.79, 0.45±0.09, 0.29±0.03, and 0.39±0.12, respectively) (P=0.062). In empty vector-transfected stem cells, the levels of these proteins prior to differentiation were 0.72±0.17, 0.81±0.11, 0.79±0.09, and 0.75±0.15, respectively; the levels of all four proteins decreased significantly following differentiation (0.32±0.09, 0.41±0.07, 0.32±0.13, and 0.36±0.06, respectively). Similar changes were observed in untransfected cells, which expressed these proteins at levels of 0.78±0.03, 0.83±0.01, 0.81±0.04, and 0.77±0.05, respectively, prior to differentiation, and at significantly lower levels following differentiation (0.31±0.11, 0.36±0.12, 0.37±0.11, and 0.38±0.02, respectively). The levels of E-cadherin (a marker of differentiation) in these cells were also determined by Western blotting. In WWOX-expressing cells, the E-cadherin level did not alter significantly (mean ± SD prior to differentiation, 0.69±0.09; following differentiation, 0.71±0.02). In empty vector-transfected cells, the E-cadherin level was 0.41±0.02 prior to differentiation, and increased significantly to 0.72±0.01 following differentiation. Similarly, the E-cadherin level was 0.46±0.06 in untransfected stem cells prior to differentiation, increasing significantly to 0.71±0.04 when the cells had differentiated (Fig. 4).

### WWOX confers sensitivity to chemotherapeutic drugs in ovarian cancer stem cells

Ovarian cancer stem cells were treated with cisplatin, doxorubicin and mitoxantrone, to evaluate their sensitivity to chemotherapeutic drugs (Table I). Under the same treatment conditions for all drugs, the relative viability of WWOX-expressing cells was significantly lower than that of empty vector-transfected cells or untransfected cells, indicating that WWOX-expressing cells had an attenuated resistance to the three drugs. When treated with 0.25 μg/ml cisplatin, the relative viability of WWOX-expressing cells was 0.593±0.136 (P=0.009), significantly lower than empty vector-transfected cells (0.823±0.103) or untransfected cells (0.861±0.201). Similarly, in the presence of 0.5 μg/ml cisplatin, the relative viability of WWOX-expressing cells (0.372±0.136) was significantly lower compared with that of empty vector-transfected cells (0.731±0.167) or untransfected cells (0.702±0.129). Treatment with 0.5 μmol/l doxorubicin significantly lowered the relative viability of WWOX-expressing cells (0.461±0.092) compared with empty vector-transfected cells (0.782±0.133) or untransfected cells (0.841±0.211). When doxorubicin was used at 1.5 μmol/l, the relative viability of WWOX-expressing cells (0.266±0.004) was also significantly lower compared with that of empty vector-transfected cells (0.601±0.007) or untransfected cells (0.582±0.121). When treated with 0.05 μg/ml mitoxantrone, the relative viability of WWOX-expressing cells (0.396±0.101) was significantly lower than either empty vector- transfected cells (0.702±0.141) or untransfected cells (0.631±0.161); when doxorubicin was used at 0.25 μg/ml, the relative viability of WWOX-expressing cells (0.103±0.136) was significantly lower compared with that of empty vector-transfected cells (0.424±0.002) or untransfected cells (0.371±0.003).

### WWOX inhibits the in vivo tumorigenicity of ovarian cancer stem cells

**Figure 5:**
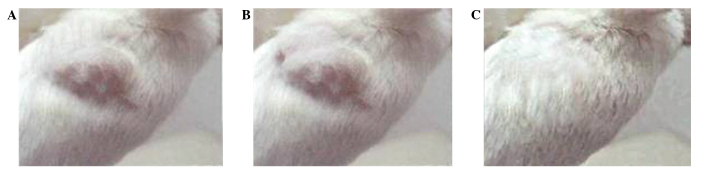
WWOX reduces the *in vivo* tumorigenicity of ovarian cancer stem cells. (A) Untransfected cells formed a tumor (43 days after the transplantation of 1×10^3^ cells); (B) empty vector-transfected cells formed a tumor (48 days after the transplantation of 1×10^3^ cells); (C) WWOX-expressing plasmid-transfected cells did not form a tumor (no tumor formation 50 days after the transplantation of 1×10^3^ cells). WWOX, WW domain-containing oxidoreductase.

When ovarian cancer stem cells were transplanted into NOD/SCID mice, empty vector-transfected cells and untransfected cells formed tumors with a minimum of 1×10^3^ cells, exhibiting a tumorigenesis rate of 2/5 and a tumor-forming time of 43–55 days for the two groups. Transplanting a greater number of cells led to an increase in tumorigenesis rate, and reduction in tumor-forming time. WWOX-expressing cells only formed tumors *in vivo* when a minimum of 2×10^4^ cells were transplanted. Compared with empty vector-transfected cells and untransfected cells at the same dose, WWOX-expressing cells had a lower rate of tumorigenesis (1/5, compared with 5/5 for empty vector-transfected and untransfected cells) and a longer tumor-forming time (76.9 days, compared with 27.3 and 28.1 days for untransfected and empty vector-transfected cells, respectively). Thus the tumorigenicity of WWOX-expressing cells was 20-fold lower than that of untransfected or empty vector-transfected cells (Table II and Fig. 5).

## Discussion

The cancer stem cell theory provides a novel perspective on the processes of tumor-initiation and progression (12). Cancer stem cells have the following properties: i) Self-renewal capability; ii) differentiation potential; iii) expression of stem cell markers; iv) resistance to chemotherapeutic drugs; and v) tumorigenicity in immune-deficient mice (13–16). In the current study, the plasmid pcDNA3.1-*WWOX* was transfected into human ovarian cancer stem cells to determine the effects of WWOX overexpression on stem cell properties.

In the sphere-forming assay, WWOX-expressing cells exhibited a significantly diminished sphere-forming ability compared with the control cells (empty vector-transfected cells or untransfected cells), indicating that WWOX expression inhibits the self-renewal capability of ovarian cancer stem cells. Conversely, when grown in serum-containing medium to induce cellular differentiation, WWOX-expressing stem cells adhered and grew more readily compared with control cells. Flow cytometry analysis revealed that, in control cells, the CD133+ and CD117+ cell populations decreased following differentiation, while fewer WWOX-expressing cells were positive for CD133 and CD117 compared with control cells prior to differentiation, and this did not alter significantly following differentiation. In addition, Western blot analysis revealed that the expression of the stem cell markers ABCG2, Nanog, OCT4, and BCRP in control cells reduced following differentiation; WWOX-expressing cells, by contrast, exhibited lower levels of these proteins prior to differentiation, and these levels did not decrease significantly following differentiation. Conversely, control cells produced more E-cadherin following differentiation, while WWOX-expressing cells produced more E-cadherin compared with control cells prior to differentiation, but exhibited no significant increase in E-cadherin production following differentiation. Collectively, the results suggest that WWOX-expressing cells lose their potential for differentiation.

Cancer stem cells differ from mature, differentiated cells in their resistance to chemotherapeutic drugs (17). In the current study, the first-line chemotherapy drug for ovarian cancer, cisplatin, and the two most widely used drugs in cancer stem cell studies, doxorubicin and mitoxantrone, were used to evaluate the alterations in drug sensitivity in WWOX-expressing ovarian cancer stem cells. Consistent with the results from other cancer types (18–22), ovarian cancer stem cells exhibited multi-drug resistance. However, when WWOX was expressed, these cells exhibited significantly decreased resistance to these drugs, indicating that WWOX may reverse the drug resistance of ovarian cancer stem cells.

Ovarian cancer stem cells were also transplanted into female NOD/SCID mice in order to determine their *in vivo* tumorigenicity. The results indicated that ovarian cancer stem cells possessed high tumorigenicity, however, WWOX expression led to a lowered tumorigenesis rate and a longer time for tumors to form, suggesting that WWOX expression reduces tumorigenicity in these cells.

In conclusion, cancer stem cells derived from the human ovarian cancer cell line HO8910 possess stem cell properties, including self-renewal ability, differentiation potential, *in vivo* tumorigenic capability, expression of stem cell markers at high levels, and multiple drug resistance. The results also indicate that WWOX expression may suppress the stem cell properties of these cells. The present study provides an experimental basis for ovarian cancer gene therapy, as the WWOX gene was found to exhibit a significant effect on the biological behavior of ovarian cancer stem cells and thus, may present a novel therapeutic target for ovarian cancer treatment. Future studies are required to elucidate the mechanisms governing WWOX-mediated inhibition of stem cell properties in ovarian cancer stem cells, and to develop WWOX gene therapy as a novel biological therapeutic method to improve survival of ovarian cancer patients.

## Figures and Tables

**Table I:** WWOX increases chemosensitivity of ovarian cancer stem cells to chemotherapeutic drugs following gene transfection.

	Relative cell viability, mean ± standard deviation					
	
	Cisplatin		Doxorubicin		Mitoxantrone	
			
Cell group	0.25 μg/ml	0.5 μg/ml	0.5 μmol/l	1.5 μmol/l	0.05 μg/ml	0.25 μg/ml
WWOX-expressing cells	0.593±0.136	0.372±0.136	0.461±0.092	0.266±0.004	0.396±0.101	0.103±0.136
Empty-vector cells	0.823±0.103	0.731±0.167	0.782±0.133	0.601±0.007	0.702±0.141	0.424±0.002
Untransfected cells	0.861±0.201	0.702±0.129	0.841±0.211	0.582±0.121	0.631±0.161	0.371±0.003

WWOX, WW domain-containing oxidoreductase.

**Table II:** WWOX reduces *in vivo* tumorigenic capability of ovarian cancer stem cells.

	Tumorigenesis rate (tumor forming time, days)			
	
Cells	10^3^ cells	2×10^3^ cells	2×10^4^ cells	2×10^5^ cells
Untransfected	2/5 (43–52)	5/5 (30–44)	5/5 (19–33)	5/5 (14–26)
Empty vector	2/5 (48–55)	5/5 (29–45)	5/5 (20–31)	5/5 (15–27)
WWOX-expressing	0/5 (N/A)	0/5 (N/A)	1/5 (68)	5/5 (73–87)

WWOX, WW domain-containing oxidoreductase; N/A, not applicable.
